# Spatial Variability of Benthic-Pelagic Coupling in an Estuary Ecosystem: Consequences for Microphytobenthos Resuspension Phenomenon

**DOI:** 10.1371/journal.pone.0044155

**Published:** 2012-08-29

**Authors:** Martin Ubertini, Sébastien Lefebvre, Aline Gangnery, Karine Grangeré, Romain Le Gendre, Francis Orvain

**Affiliations:** 1 Université de Caen Basse-Normandie, FRE3484 BioMEA, Caen, France; 2 CNRS INEE, FRE3484 BioMEA, Caen, France; 3 IFREMER, LERN, Port en Bessin, France; 4 Université de Lille1, UMR CNRS 8187 LOG “Laboratoire d’océanologie et geosciences”, Station Marine de Wimereux, Wimereux, France; 5 CNRS, UMR 7208 BOREA, Muséum d’histoire naturelle, CRESCO, Dinard, France; National Institute of Water & Atmospheric Research, New Zealand

## Abstract

The high degree of physical factors in intertidal estuarine ecosystem increases material processing between benthic and pelagic compartments. In these ecosystems, microphytobenthos resuspension is a major phenomenon since its contribution to higher trophic levels can be highly significant. Understanding the sediment and associated microphytobenthos resuspension and its fate in the water column is indispensable for measuring the food available to benthic and pelagic food webs. To identify and hierarchize the physical/biological factors potentially involved in MPB resuspension, the entire intertidal area and surrounding water column of an estuarine ecosystem, the Bay des Veys, was sampled during ebb tide. A wide range of physical parameters (hydrodynamic regime, grain size of the sediment, and suspended matter) and biological parameters (flora and fauna assemblages, chlorophyll) were analyzed to characterize benthic-pelagic coupling at the bay scale. Samples were collected in two contrasted periods, spring and late summer, to assess the impact of forcing variables on benthic-pelagic coupling. A mapping approach using kriging interpolation enabled us to overlay benthic and pelagic maps of physical and biological variables, for both hydrological conditions and trophic indicators. Pelagic Chl *a* concentration was the best predictor explaining the suspension-feeders spatial distribution. Our results also suggest a perennial spatio-temporal structure of both benthic and pelagic compartments in the ecosystem, at least when the system is not imposed to intense wind, with MPB distribution controlled by both grain size and bathymetry. The benthic component appeared to control the pelagic one via resuspension phenomena at the scale of the bay. Co-inertia analysis showed closer benthic-pelagic coupling between the variables in spring. The higher MPB biomass observed in summer suggests a higher contribution to filter-feeders diets, indicating a higher resuspension effect in summer than in spring, in turn suggesting an important role of macrofauna bioturbation and filter feeding (*Cerastoderma edule*).

## Introduction

Estuaries are known to be among the most productive systems in the biosphere [1]. Their high productivity is mainly due to the presence of nutrients and of multiple food resources for the trophic web, coming from both riverine, marine planktonic and benthic compartments [2]. Moreover, in most of these shallow water environments, the intensity of the physical factors reinforces the connections between benthic and pelagic environments by increasing material processing, nutrient cycling and erosion/deposition exchanges. Among all these processes, microphytobenthos (MPB) resuspension is a major phenomenon involved in benthic-pelagic coupling since MPB can contribute up to 50% or more of the primary production for such ecosystems [3]. Consequently, MPB resuspension has major implications both for the food web and for ecosystem stability [4], [5] [6].

**Figure 1:**
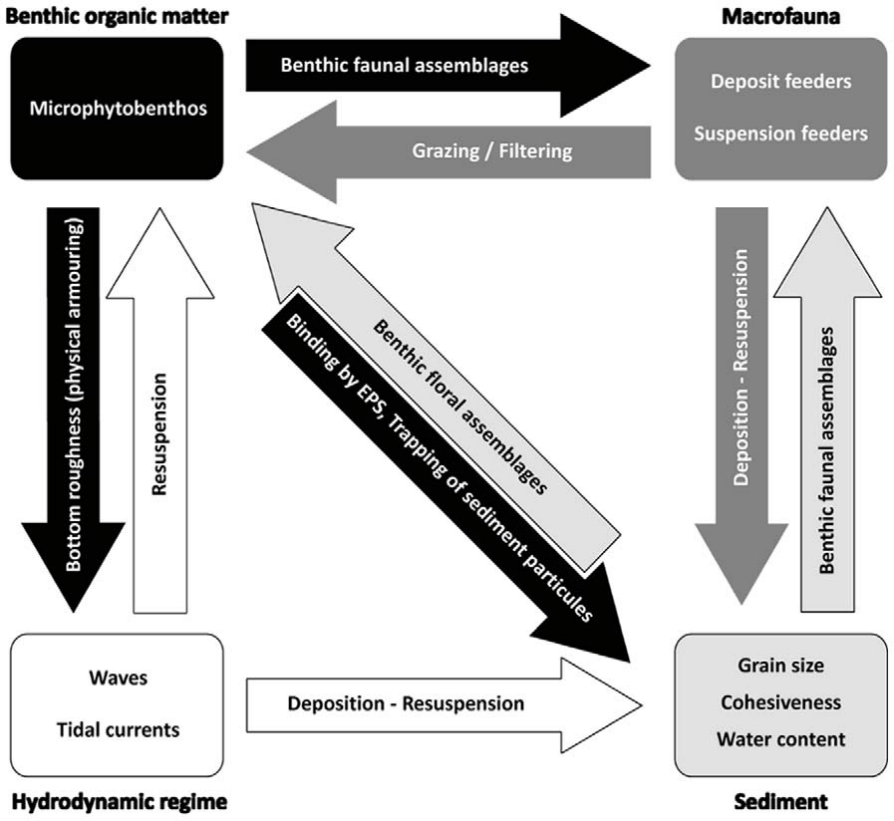
Factors involved in sediment resuspension and the associated microphytobenthos.

Benthic-pelagic coupling and especially MPB resuspension are controlled by a complex set of interactions (Fig. 1) between biological, physical, and chemical components or processes [7]. Physical processes such as waves and tidal currents are responsible for erosion of the sediment, leading to sediment resuspension in the water column [8], and hence modifying the properties of the sediment. These mechanisms directly control sediment erodibility, especially sediment composition and compaction [9]. The associated MPB is resuspended at the same time, with wind effect being the major physical factor controlling its resuspension [4]. Even if MPB resuspension is directly controlled by bulk sediment properties related to erodibility, MPB is partly able to control its own resuspension behavior by producing exopolymeric substances (EPS), which reinforces the surface cohesion [10], [11], [12]. This biofilm structure may also cause physical armoring of the sediment, thus limiting its erosion [7]. Macrofauna may also affect the resuspension of MPB by bioturbation, affecting sediment erodibility by 1) releasing a material with a high concentration of microphytobenthos [13] and 2) reducing MPB biomass due to nutrition [14]. As a consequence of trophic interactions, MPB can influence long-term trends in benthic macrofauna composition [15], which in turn influence differently MPB resuspension by bioturbation.

MPB biomass always varies in space and over time at all scales in the sedimentary landscape. For instance, surface MPB biomass can double at a given site within one day [16], and MPB biofilms also oscillate in response to the tidal 14-day cycle [17], [18]. In intertidal areas, MPB biomass also varies with the season, and the lowest and highest biomass are found in winter and summer, respectively [16], [19]. Variation in MPB biomass from one year to the next appears to be low [19]. Physical variables such as light irradiance [20], temperature [21], nutrient concentration [22] or wind intensity [19], may be responsible for these seasonal variations in MPB biomass, and many of these time forcing variables can also cause variations at different spatial scales. Even if irradiance varies over the year, light availability is closely related to the bathymetry, and thus influences benthic production [18], [19]. Grain-size is not homogenous within the intertidal area, leading to differential distribution of sediments with different degrees of erodibility, and sediment composition could be the main factor that regulates the spatial patterns of MPB biomass [17]. These differences between sediment types lead to different diatom assemblages with epipsamic or epipelic diatoms causing related variation in MPB biomass. If the previously described physical variables act as a bottom-up control of MPB biomass [17], biological phenomena such as grazing can act as a top-down control [23]. Even if the spatio-temporal dynamics of microphytbenthos production and biomasses are now better understood, the extent to which the MPB biomass supplies the water column is poorly described and quantified.

Different approaches have been used to characterize benthic-pelagic exchanges caused by MPB resuspension phenomenon. This phenomenon has been widely studied using flume experiments, which enable quantification of the relationship between bed erodibility and sediment properties [24]. However, flume studies focus on the initial point of the erosion phenomenon, without tracking the source distribution of particles along Lagrangian movements of water bodies. Most of the time those data are used for model parameterization for a further evaluation of its fate in the water column. Although flume and small mesocosms experiments are useful to quantify resuspension rates at small scales, they do not enable assessment of the implications of resuspension processes for benthic-pelagic coupling and trophic redistribution at the ecosystem scale [25]. Bivalve farmings have often been recognized as habitats where microphytobenthic communities colonize rapidly the sediments in relation to deposit and bed flow properties mediation by the effect of farming structures and alimentary behavior of animals [17], [25].

Different proxies have been used to study the benthic-pelagic coupling and they can be used as well to better define the trophic routes of resuspended microphytobenthos within an ecosystem. Chlorophyll *a* (Chl *a*) biomass, which is often used as a proxy for phytoplankton biomass [26], can also be used as a proxy for resuspension [27], but this variable include both benthic and pelagic sources of Chl *a*. The taxonomic ratio of benthic to pelagic microalgae can be used as a quantitative indicator for resuspension phenomena [28], thus refining the Chl *a* concentration indicator. However, differences between benthic and pelagic diatoms are not that obvious since some species are tychopelagic, i.e. live in both environments. Like for Chl *a* concentration, particulate suspended inorganic matter (SPiM) can be a good indicator of resuspension if both benthic and pelagic compartments are studied at the same time, but the time lag is difficult to avoid *in situ*, particularly when a whole ecosystem is being studied. Some authors used isotopic signatures with δ^13^C and δ^15^N values of suspension feeders to determine the MPB contribution to their diets [29], [30], [31]. In fact, they could be indirectly used as a proxy of amounts of resuspended MPB, but isotopic studies focus on the final point, i.e. consumption, without knowing whether the initial MPB primary production was autochthonous or allochthonous. Such information could be very useful to consider for coastal management and ecological implications in terms of habitat connection and trophic interaction. The use of phaeopigments as a grazing indicator has been discussed by several authors and judged to be useful for studies of the water column [32], [33]. Because *in situ* studies include many parameters and all these indices provide substantial information concerning different aspects of benthic-pelagic coupling, the combination of them is the best way to assess the implication of MPB resuspension and its redistribution in the pelagic ecosystem and along the trophic chain.

Understanding the set of multifactorial interactions at the ecosystem scale is of critical importance to quantify exports of MPB to the water column, its relative importance compared to the phytoplankton communities and to hierarchize the physical and biological factors potentially involved in MPB exportation. To our knowledge, field experiments have never included both benthic and pelagic compartments at a large scale to explore MPB resuspension phenomenon even though they are complementary and very difficult to separate in estuaries. Because MPB is simultaneously consumed and exported to the water column, in this study we overlaid benthic and pelagic maps of physical and biological variables, for both hydrological conditions and trophic indicators.

The multiple criteria approach we used to study the indices at all scales enabled us to explain the resuspension within the whole ecosystem approach and to cope with the absence of flux measurements (i.e. erosion as well as trophic fluxes). This study also included a spatial survey of MPB distribution, the factors explaining its resuspension and finally its consumption by filter feeders. To better assess the temporal variations in benthic-pelagic coupling, benthic and pelagic compartments were studied simultaneously at two contrasted seasons in terms of forcing variables and MPB and phytoplankton biomass within a temperate macrotidal and exploited coastal ecosystem, the “Baie des Veys” (BDV, France). The whole intertidal area was sampled to account for the spatial heterogeneity within the Bay including different spatial patterns of forcing factors (presence/absence of shellfish farmings, sediment composition, macrofauna distribution, bed shear stress, salinity). Concerning temporal variability, MPB production is normally low in early spring and high in late summer, but the spring phytoplankton bloom is normally higher than the late summer bloom, so resuspension and its relative contribution as a trophic resource in the water column is expected to be higher in late summer. Bioturbation activities that could lead to the resuspension of microphytobenthos from intertidal sediments are also expected to be amplified at the end of summer because of the high levels of biomass but also because of the positive effects of temperature.

## Materials and Methods

### 1. Study Area

**Figure 2:**
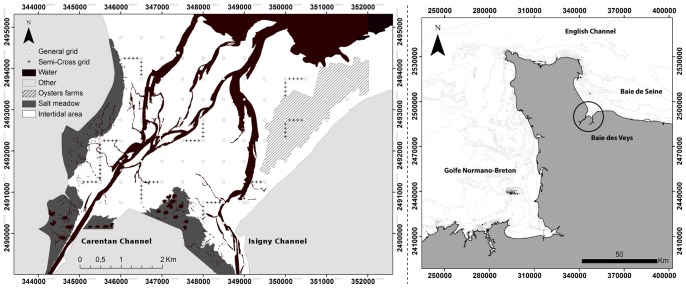
Location of the Baie des Veys and sampling grid.

The *Baie des Veys* (BDV, Fig. 2) is an estuarine bay located in the western part of the Bay of Seine in the eastern English Channel. It is characterized by an intertidal area covering 37 km^2^ and a macrotidal regime that reaches 7 m maximum tidal amplitude during spring tides and 2.5 m during neap tides [34]. The bay is quite well protected from the prevailing wind by the Cotentin peninsula. Current velocity can reach 3 m.s^−1^ during flood tides and 1.5 m.s^−1^ during ebb tides [29]. Four rivers flow into the BDV through two channels, the Isigny channel in the east and the Carentan channel in the west. Freshwater runoff is low in summer and high in winter, with flows ranging from 3.7 to 26.4 m^3^.s^−1^ in the Carentan Channel and from 23.9 to 40.4 m^3^.s^−1^ for the Isigny Channel. The oyster farming area extends into the north-eastern part of the bay.

### 2. Sampling Strategy

Both benthic and pelagic variables were sampled during spring tides to better assess the contribution of resuspended MPB to the total Chl *a* content in the water column [4]. Benthic samples were collected within a week between March 29 and April 2, 2010, and water samples were collected on April 29 and 30, 2010. For the 2 sampling periods, the tidal amplitude was approximately 5.5 m. The same strategy was applied at the end of summer, to assess the impact of the increased number and activity of mollusks on the resuspension phenomenon. Benthic samples were collected from September 8 to 12, 2010, and water samples on the 13 and 14 of September. Because farming structures are located in the north-eastern part of the bay, they may benefit from both autochtonous and allochtonous resources coming from the south of the bay. As a consequence, water was sampled during spring ebb tides, to account for the flux of MPB from the southern part to the north part of the bay, potentially feeding the suspension-feeders reared in the bay. A systematic grid of 88 points was extended over the entire map of the intertidal area with a sampling interval of 500 m (Fig. 2). Heterogeneity in soil organism distribution occurs at nested scales, and is shaped by a spatial hierarchy of environmental factors, intrinsic population processes and disturbances [35]. To explore smaller scale distributions, a nested sampling design was applied [36], [17]. The intertidal area was divided into three sub-domains that were considered as distinct areas due to their separation by the Isigny and Carentan Channels. In each sub-domain, a semi-cross sampling design was applied with an interval of 100 m between each point (Fig. 2). Each semi-cross was placed on high gradient areas previously observed on the field [17].

### 3. Field Measurements

#### Sediment and benthos sampling

At low tide, four 20 cm diameter cores were collected at each sampling site. The first cm of each core was removed and placed in a separate plastic bag. At each site, macrofauna were harvested within a square of 0.25 m^2^. The choice of a surface of 0.25 m^2^ is appropriate when referring to several studies carried out on the benthos in the intertidal zone showing that this surface is sufficiently large and well suited for estimating the abundance of bivalves [37]. This surface allows a suitable and satisfactory sampling of the fauna whatever its distribution (contagious, regular, or random), even when populations are small in number [38].

The entire sediment was sampled to a depth of 10 cm by hand, and then sieved directly on the spot using a 1 mm mesh size sieve [39]. The depth of 10 cm was chosen in order to take in account most of the mollusk biomass potentially involved in resuspension phenomenon, assuming the fact that some bivalve species like *Mya arenaria* that was observed on the field live under this depth [40]. Sieved samples were placed in plastic bags for transport to the lab.

#### Animal sampling for isotopy

Six sampling sites were selected within the farming structures to assess the spatial variability of the suspension-feeder *Crassostrea gigas* diet by sampling the widest extent of the structures. The bathymetry of all sites was between +1.65 and +3.50 m. Sites 1 and 2 were on hard substrata, and other sites were on soft-bottom. At each sampling site, five oysters were sampled in the two seasons one month after the spring and late summer samplings, in order to investigate their diets by stable isotopic ratios of carbon and nitrogen.

#### Water column sampling

Surveys were carried out during ebb tide at all grid points, from one hour after high tide to one hour before low tide. For the 2 periods, the wind velocities were found to be relatively low and similar (3.44 and 4.67 m s^−1^ for April and September respectively), as well as the wind direction with dominant west-northwestern winds (213.33 and 251.46° in a 360° compass rose for April and September respectively). At each point, 5 L water samples were collected by pumping water at a height of 1 m above the seafloor, to ensure access to resuspended MPB. Water samples passed directly through a home-made device equipped with a multi-parameter sensor YSI 6600 (YSI, Yellow Springs, Ohio, USA), before being stored for laboratory measurements. Water subsamples were immediately preserved in Lugol solution for the determination of flora.

### 4. Laboratory Analyses

#### Sediment

Back at the laboratory, sediment samples were pooled and mixed thoroughly, and a 1.5 ml subsample was removed and stored at −20°C in the dark until Chl *a* analyses. The remaining sediment was also stored at −20°C until grain-size measurements. Each subsample of sediment was freeze-dried and then weighed to determine the sediment water content. Chl *a* was measured on freeze-dried subsamples using a fluorometric method to estimate algal biomass (µg.g^−1^ sediment). The Chl *a* content of the sediment was extracted in 90% acetone at 4°C for18 h in the dark. The chlorophyll extracts were measured after centrifugation on a Turner Designs TD 700 fluorimeter (USA) following the method of Welschmeyer [41]. Analysis of particle size distribution was performed by using a grain-size laser method. Sediment samples were dried at 60°C for 3 days and sieved (for coarse-grained particles >2000 µm). Organic matter was removed from the samples with H_2_O_2_, followed by soil dispersion with sodium hexametaphosphate. Then, grain-size analysis was performed using a laser granulometer (Coulter, LS200, USA). For the sake of simplicity, the size fractions obtained using the Wenworth scale were then classified in two groups: mud (0–63 µm) and sand (63–2000 µm).

#### Macrofauna

Samples were fixed in a 10% formaldehyde solution for 24 h and transferred to 70% ethanol for storage until further analyses. All samples were carefully sorted to separate organisms and the remaining sediment. The mollusk species were then determined [42]. Mollusk flesh was separated from the shell, dried at 60°C for 3 days and weighed without the shell. Small specimens with a tough shell (e.g. *Peringia ulvae*) were treated with a drop of 33% hydrochloric acid solution for a few minutes to dissolve the shell. The organisms were then dried in an oven at 450°C for 4 hours to obtain the ash free dry weight.

Freeze–dried, powdered, and homogenized oyster samples were analyzed using a CHN elemental analyzer (EuroVector, Milan, Italy) for particulate organic carbon (POC) and particulate nitrogen (PN) in order to calculate their C/N atomic ratio (Cat/Nat). The analytical precision of the experimental procedure was estimated to be less than 2% DW for POC and 6% DW for PN. The gas resulting from the elemental analyses was introduced online into an isotope ratio mass spectrometer (IRMS) (GV IsoPrime, UK) to determine carbon and nitrogen isotopes. Stable isotopic data are expressed as the relative per mil (‰) differences between the samples and the conventional standards, Pee Dee Belemnite (PDB) for carbon and atmospheric N_2_ for nitrogen, according to the following equation:
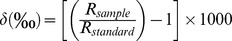
where *δ* is ^13^C or ^15^N abundance and *R* is the ^13^C:^ 12^C or ^15^N:^14^N ratio. The internal standard was the USGS 40 of the International Atomic Energy Agency (*δ* ^13^C = −26.2; *δ* ^15^N = −4.5). The typical analytical precision was ±0.05‰ for carbon and ±0.19‰ for nitrogen. The Phillips and Gregg mixing model [43] was used to estimate spatio-temporal variations in the contribution of suspended organic matter (OMS), including particulate organic matter (POM), MPB, resuspended POM (rPOM), and macroalgae (ULV), to the suspension-feeders’ diets, following the protocol of Lefebvre et al. [7] but with fractionation values of 1.85‰ for *δ* ^13^C and 3.79‰ for *δ* ^15^N, obtained from Dubois et al. [44].

#### Water samples

To measure the concentration of suspended particulate matter, two subsamples (1L) were sieved and passed through weighed and dried glass-fiber filters (Whatman GF-C), washed with distilled water to avoid errors due to salt, packed in petrislides (Millipore, USA), and immediately stored at −20°C until analyses. The filters were dried in an oven at 60°C for 72 hours. For Chl *a* concentration measurements, two subsamples were sieved and passed through a glass-fiber filter (Whatman GF-C), folded and placed in a tube at −20°C before analyses. The Chl *a* content was extracted in 90% acetone for 18 h at 4°C in the dark. After short centrifugation (3500 G), the chlorophyll extracts were measured on a Turner Designs TD 700 fluorimeter (USA) following the method of Welschmeyer [41] and expressed as chlorophyll content (µg.L^−1^) in the spring samples. The summer samples were analyzed using Lorenzen’s method [45] in order to examine the phaeopigment content. Calibration was performed between the two methods to compare the result of the two samplings (y = 0.9624x+1.5399, R^2^ = 0.999). Each sample preserved in Lugol was observed for quantitative/qualitative determination of microalgae flora, following the Utermohl method described in [46] using light microscopy on Sedgewick-Rafter cells. In some samples, 400 individual cells were counted whatever the total number of cells, following the European standard for phytoplankton counting (NF EN 15204, 2006). Finally, a list of diatoms and the ratio of benthic to pelagic diatom species were established for each site following the protocol of Kasim and Mukaï [28]. Actually, the quantity of larger species is underestimated using abundances, while of the smaller species is underestimated using biomass [47]. To get round this problem, log-transformed abundance scores were used to calculate this ratio [48].

### 5. Statistical Analyses

Geostatistical analyses were performed with the ArcGIS extension Geostatistical Analyst (ESRI, USA) in order to map the different variables measured on the field. Since there was a high spatial dependency in all the variables measured, kriging was chosen as the best interpolation method to predict values for the whole intertidal area. Normal distribution was checked before each analysis and log-transformation was applied as a function of the variable concerned. Global trends were also examined, to enable removal of the possible effect of the tidal circulation on the water column. If necessary, detrending was applied using a polynomial algorithm of chosen order. Each variable was studied to find the best semivariogram model fitting for data, between circular, spherical, exponential and gaussian models (Table 1). Cross-validation enabled us to check the validity of the semi-variogram models we selected. Nugget effect was always small and never reached up to 1/3 of the sill value (Table1), confirming the validity of the sampling scale and chosen nested design scale. If the prediction errors are not biased, the mean prediction error should be near zero. However, this value depends on the scale of the data; to standardize these, the standardized prediction errors give the prediction errors divided by their prediction standard errors. The mean of these, called “mean standardized”, should also be near zero. If the prediction standard errors are valid, the root mean squared standardized error should be close to 1. If it is greater than 1, the variability of the predictions has been underestimated, and inversely. The MARS-3D hydrodynamic model [49] was used to obtain the mean bottom current velocities at the two sampling periods. The results were plotted using the ArcgiS Toolbox “MGET” [50].

**Table 1:** Variogram models with their parameter values and cross-validation results.

Spring sampling						
Variable	Benthic Chl *a*	Mud fraction	Macrofauna biomass	Pelagic Chl *a*	SPiM	Salinity
**Kriging type**	Ordinary	Ordinary	Ordinary	Universal	Universal	Ordinary
**Detrending order**	None	None	None	First	First	None
**Transformation**	Log	Log	Log	Log	Log	None
**Variogram model**	Spherical	Gaussian	Exponential	Circular	Circular	Gaussian
**Anisotropy**	True	True	False	False	True	True
**Nugget**	0.132	0.469	0.080	0.136	0.163	1.655
**Sill**	0.894	1.460	1.232	0.419	0.489	6.953
**Range**	3122.358	2937.798	968.969	1273.001	423.666	3078.753
**R^2^ (variogram)**	0.991	0.987	0.881	0.925	0,732	0,857
**Mean std.**	0.016	0.000	−0.024	−0.025	−0.046	-0.005
**RMSS**	0.951	1.198	0.968	1.122	0.946	1.054
**Summer sampling**						
**Variable**	**Benthic Chl ** ***a***	**Mud fraction**	**Macrofauna biomass**	**Pelagic Chl ** ***a***	**SPiM**	**Salinity**
**Kriging type**	Ordinary	Ordinary	Ordinary	Ordinary	Ordinary	Ordinary
**Detrending order**	None	None	None	None	None	None
**Transformation**	Log	None	Log	Log	None	Log
**Variogram model**	Gaussian	Circular	Exponential	Circular	Exponential	Spherical
**Anisotropy**	False	True	False	False	False	False
**Nugget**	0.319	37.4	0.040	0.037	0.865	0.002
**Sill**	0.677	101	1.46	0.174	18.0	0.347
**Range**	2242	3456	1101	2675	2714	2568
**R^2^ (variogram)**	0.841	0.730	0.924	0.965	0.847	0.934
**Mean std.**	−0.002	−0.017	−0.050	−0.023	0.001	−0.022
**RMSS**	1.06	1.067	1.178	1.068	1.172	0.943

Mean std  =  Mean standardized; RMSS  =  Root Mean Square standardized.

Multivariate analysis using the R package ADE4 (R-project) were used to better identify spatial and seasonal effects and explore the benthic-pelagic coupling through correlations between the variables. Principal Components Analyses (PCA) were performed on benthic and pelagic log-transformed datasets for both seasons, completed with estimated data from the kriging matrices for the few numbers of points where there were some missing values. For these analyses, bathymetry was considered as an auxiliary variable because it can play a role in both benthic and pelagic compartments. Co-inertia analysis was used to explore the relationships between the benthic and pelagic compartments by coupling the previous PCA, and its validity was checked by performing a Monte-Carlo test on the sum of eigenvalues of the analysis [51]. Frequency distribution of the RV values for 100 random co-inertia simulations was tested to check the validity of the co-inertia analyses.

Regression analyses were performed using Minitab (Minitab inc., USA) in order to find the best model predicting variable distribution. Stepwise regression was used to identify the best subsets of predictors in sampled variables. A linear regression was then applied on the most appropriate subset of data, corresponding to the best Awaike Information Criterion (AIC), meaning the lowest values when comparing the different regression models.

## Results

### Benthos

**Figure 3:**
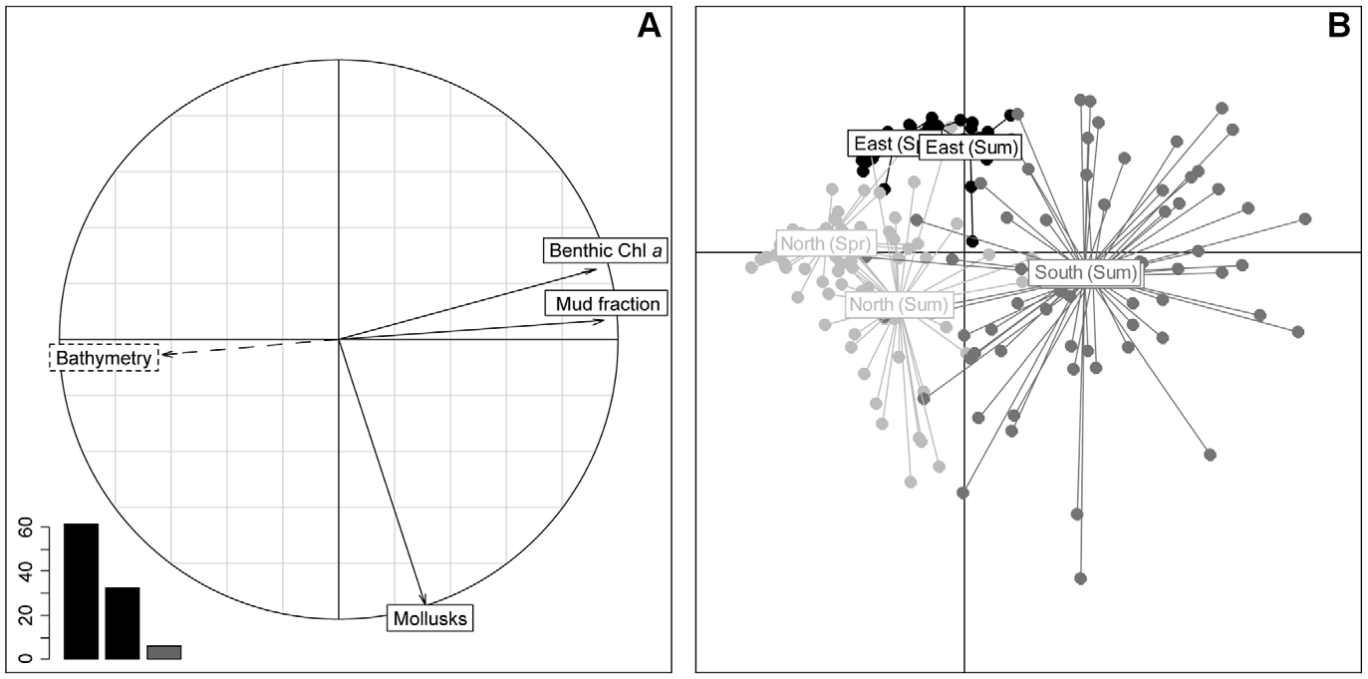
PCA results of the benthic log-transformed variables for the 2 seasons. Bathymetry (m), Chl *a* concentration (µg.g^−1^), mud fraction (% of total sediment) and mollusk biomass (g AFDW.m^−2^). Data used for the PCA resulted from the extraction of the corresponding kriged maps on the general sampling grid. Bathymetry was used as an auxiliary variable. A: Correlation circle; B: Scatter plot of individuals, “South (Spr)” and “South (Sum)” captions are confounded.

Data for each measured variable were analyzed using a PCA (Fig. 3). Only the two first components were kept, and these explained 93.7% of the total variation. The correlation circle (Fig. 3A) showed a clear relationship between the mud fraction, Chl *a* content and the bathymetry of the intertidal area. These three variables were well represented in the 1^st^ axis and explained 61.4% of the total variation, confirming that the distribution patterns for both the mud fraction and the Chl *a* concentration remained stable between the two seasons. Mollusk biomass distribution was not correlated with the above variables; it was well represented on the 2^nd^ axis and explained 32.3% of the total variance.

**Figure 4:**
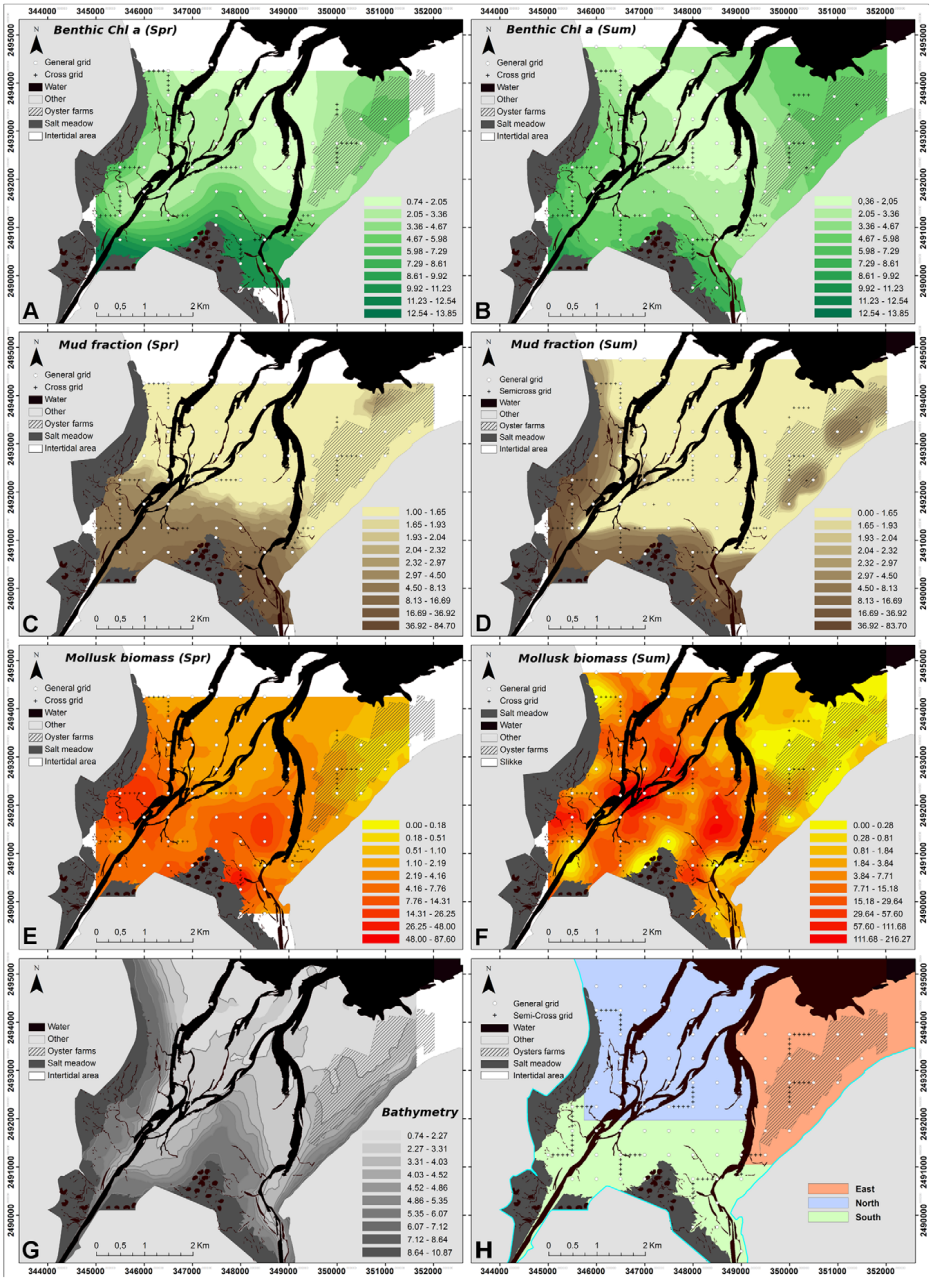
BDV spring and summer kriged maps of benthic variables for the 2 seasons. All variables were kriged on different variogram models depending on the data (Table 1). Geometrical scales were used to maximize the visualization of both gradients and the patchiness of the different variables. Mollusk maps are at different scales to account for the discrepancy in the data between the 2 sampling campaigns. **A, B**: Chl *a* concentration (µg.g^−1^); **C, D**: Mud fraction (%<63 µm of total sediment); **E, F**: Mollusks biomass (g AFDW g.m^2^). **G**: Bathymetry of the BDV, from low to high tide spring tide levels (m). **H**: Representation of the 3 subdomains defined by the PCA.

The scatter plot of individuals (Fig. 3B) showed a clear spatial structure, and sampling points were merged into three groups, corresponding to three areas of the bay: the eastern part located on the east side of the Isigny channel, and the northern and southern areas to the west (Fig. 4H). Individual distribution was explained by the correlation circle, with a Chl *a* concentration gradient from north to south, and an eastern area with a lower mollusk biomass.

Geostatistical analysis and kriged maps confirmed that benthic Chl *a* concentration (Fig. 4A, B) was characterized by the same distribution patterns at the two seasons, with higher concentration close to the salt meadow particularly at the southern borders of the bay, and also under the farming structures in the east. Less concentration was found in the central and northern part of the bay, resulting in a decreasing gradient from the coast to the center of the bay. Regarding to the three areas determined by the PCA, the southern and eastern areas were characterized by relatively high Chl *a* concentration compared to the northern area. The mud fraction (Fig. 4C, D) was correlated with the previous parameter with a gradient from the southern part of the bay with muddy to mixed sediment to sandy areas in the north and east. There was a slight increase in the mud fraction in two patches in the eastern part of the bay sampled during summer. The Chl *a* concentration and mud fraction were both clearly linked to the bathymetry of the bay (Fig. 4G), with the shallower parts located close to the salt meadow and along the eastern coast. Ordinary kriging was required for the Chl *a* concentration and mud fraction (Table 1), and the variogram structure was close considering the range (ca. 3000 m), reflecting a similar patch size for these two variables.

In contrast, there was a change in mollusk biomass between the two sampling periods (Fig. 4E,F). Five major species were identified at each season, with the cockle *Cerastoderma edule* as the dominant species (Table 2). Only one of the major species changed between the two seasons: *S. plana* was present in spring but replaced by *Abra tenuis* in late summer (Table 2). Mean mollusks biomass increased 20-fold between the two seasons (Table 2). Despite these differences, the distribution type of mollusks remained the same for the two seasons since the variogram structure was similar in terms of kriging method (Ordinary), in terms of variogram model (Exponential) and range values (ca. 1 km). Maximum biomass increased 3-fold between the two sampling dates, from 87.6 g.m^2^ in spring to 216.3 g.m^2^ in summer. The spring map shows the three-parted intertidal area, with a high mollusk biomass in the south, a lower biomass in the north and a very low biomass in the east. The summer map shows a larger high biomass area, and a contrast between the eastern part with low biomass and the northern and southern areas characterized by high biomass. Two high biomass patches were present in spring, and were still present but far bigger in summer. Very low mollusk biomass was found under the farming structures in the east at both sampling dates.

**Table 2:** Mean weight and number of mollusks per m^2^ in the 2 samplings.

Species	*C. edule*		*M. balthica*		*A. tenuis*		*H. ulvae*		*S. plana*	
Mean	ind.m^2^	g. m^2^	ind. m^2^	g. m^2^	ind. m^2^	g. m^2^	ind. m^2^	g. m^2^	ind. m^2^	g. m^2^
Spring	16.6	2.99	2.172	0.064			102	0.077	10.7	0.035
Summer	169	65.6	4.331	0.632	6.55	0.067	148	3.18		

Regression analysis (Table 3) revealed that benthic Chl *a* concentration can be predicted by the whole set of benthic and pelagic variables in spring and mud fraction, bathymetry and water Chl *a* concentration in summer. For both seasons, mollusk biomass was best predicted by the association of chl *a* water and bathymetry, with a better model adjustment for spring (R^2^ = 0.48).

**Table 3:** Response of selected variables to log-transformed benthic and pelagic variables.

Variable	Linear predictor	R^2^_adj_	AIC
Benthic Chl a (SPR)	−2.60 −0.145 mollusks*** +0.546 mud fraction*** −0.513 bathymetry*** +0.319 SPiM* −0.235 chl *a*water** +2.26 salinity*	0.78	−211
Benthic Chl a (SUM)	0.943+0.325 mud fraction*** - 0.143 bathymetry* −0.346 chl *a* water***	0.76	−257
Mollusks (SPR)	0.339+0.725 chl *a* water*** −0.624 bathymetry***	0.48	−35.3
Mollusks (SUM)	−1.46+1.81 chl *a* water*** +0.826 bathymetry***	0.13	64.5

All variables included in linear predictor are significant (*p<0.05, **p<0.01, ***p<0.001). R^2^ represents the percentage of response variable variation that is explained by its relationship with one or more predictor variables, adjusted for the number of predictors in the model. AIC (Akaike information criterion) is a measure of the relative goodness of fit of the models, best models (lower values) were kept.

### Pelagos

**Figure 5:**
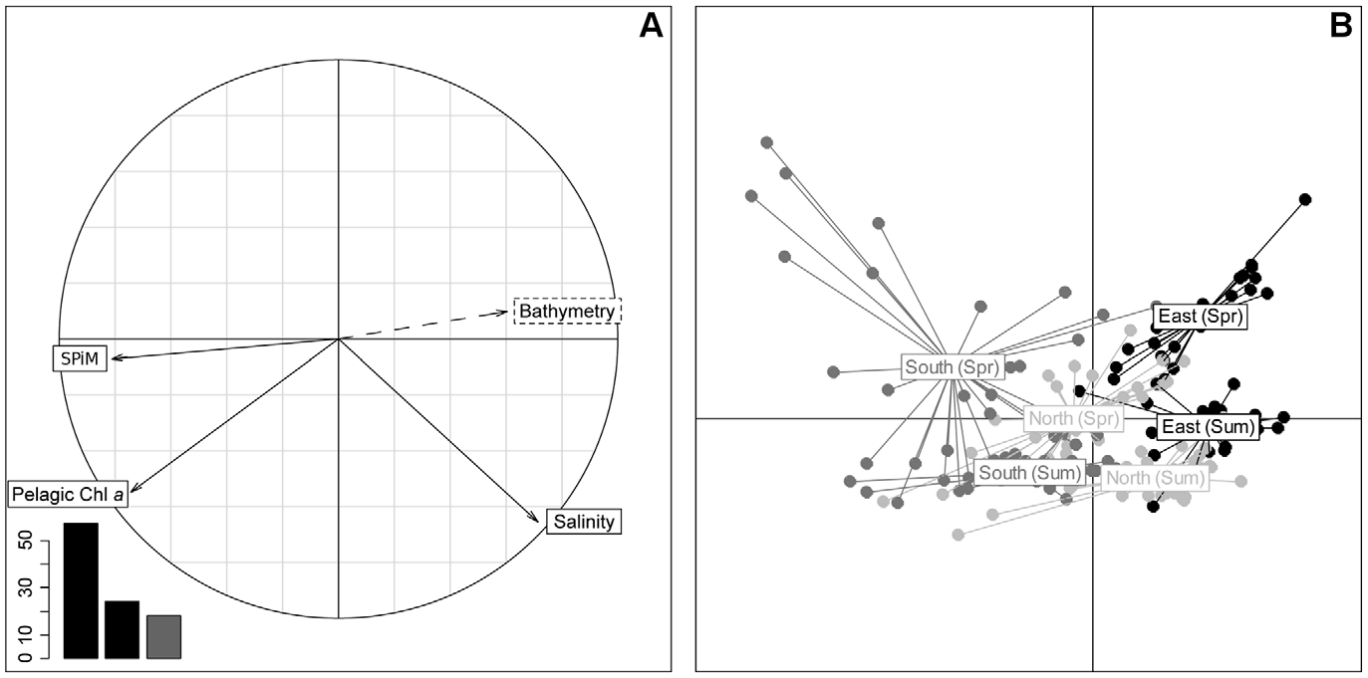
PCA results of the pelagic log-transformed variables for the 2 seasons. Bathymetry (m), Chl *a* concentration (µg.L^−1^), SPIM (mg.L^−1^) and salinity. Data used for the PCA resulted from the extraction of the corresponding kriged maps at the location of the general sampling grid. A: Correlation circle; B: Scatter plot of individuals (Spr  =  Spring; Sum  =  Summer).

A PCA was applied to the pelagic variables and only the two first components were retained, which explained 81.6% of the total variation. The correlation circle (Fig. 5A) shows that SPiM was anti-correlated with the bathymetry and was well represented on the 1^st^ axis, where it explains 57.3% of the total variation. There was also a good correlation between the pelagic Chl *a* concentration and the concentration of SPiM, even if the former was partly represented on the 2^nd^ axis. Salinity was not correlated with the pelagic Chl *a* concentration, and was represented to the same extent on both axes, but poorly anti-correlated with the concentration of SPiM. This low or null relationship between salinity and both pelagic Chl *a* concentration and SPiM concentration showed that these two variables were not entirely related to the river inlets.

In line with the results for benthos, the scatterplot of individuals (Fig. 5B) showed a clear spatial structure in the data: the sites were merged into three groups, corresponding to three spatial areas within the bay: eastern, northern and southern areas. The drift observed in the scatterplot of individuals between the two seasons was the same in all parts of the bay and appeared to be related to salinity. The southern area was characterized by the highest pelagic Chl *a* concentration while the eastern area had the lowest.

**Figure 6:**
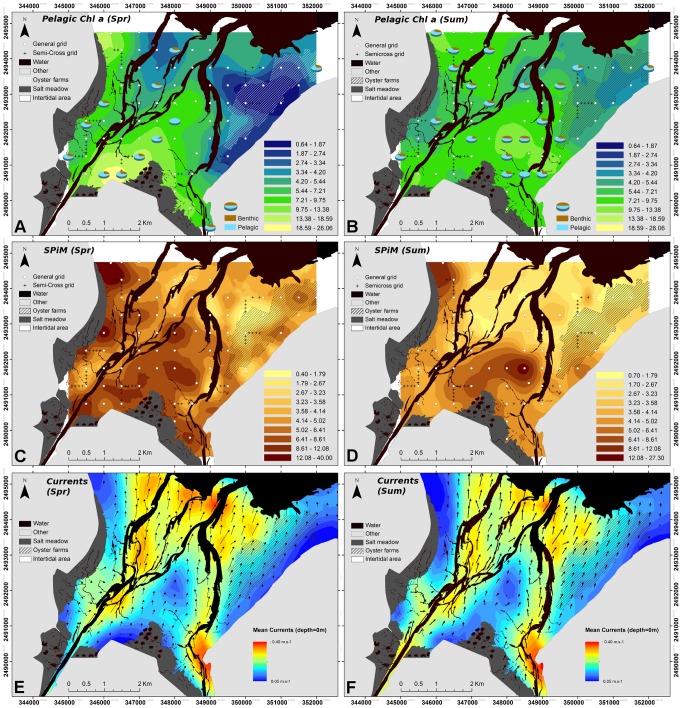
BDV spring and summer kriged maps of pelagic variables for the 2 seasons. All variables were kriged on different variogram models depending on the data (Table 1). Geometrical scales were used to maximize the visualization of both gradients and the patchiness of the different variables. Mollusk maps are at different scales to account for the discrepancy in the data between the 2 samplings. **A, B**: Chl *a* concentration (µg.L^−1^); **C, D**: SPiM amount (mg.L^−1^). **E, F**: Bottom mean current velocities and direction at the 2 sampling periods, calculated by the MARS-3D hydrodynamic model.

Kriged salinity maps (not shown) showed a common structure with a south to north gradient from low to high salinity. Salinity was twice lower in spring with stronger river inputs, particularly from the eastern channel. The southern part of the bay was characterized by high Chl *a* concentration and SPiM (Fig. 6), whereas the northern area showed lower concentrations. Both sampling periods were characterized by a depletion observed in the eastern area, which was stronger in spring. In late summer, Chl *a* concentration ranged from 2.78 to 18.8 µg.L^−1^, and the area was smaller than that found in April (from 0.64 to 26.1 µg.L^−1^). Like on the spring map, on the summer map, a large area at the north-east was characterized by a limited depletion of Chl *a* concentration in the water column. The quantity of SPiM was higher in the southern and north-western part of the bay than in the eastern part. Except in the area with the farming structures where SPiM was low, concentrations were related to the bathymetry of the bay, with lower concentrations in areas with deeper water. Mean currents at the bottom showed velocities of between 0.05 and 0.40 m.s^−1^, with higher velocities along the two channels. The two sampling periods showed similar hydrodynamic conditions with a general field of current vectors oriented towards the north-north-west of the bay during this ebb tide causing the bay to empty. A small area with lower velocities was observed in the center of the bay.

### Benthic-Pelagic Coupling

In order to examine the benthic-pelagic coupling at the bay scale, co-inertia analysis was performed on both benthic and pelagic variables. Correlation circles revealed the close link between the co-structure described by co-inertia axis F1/F2, and the structure of each dataset described by the respective components in each PCA. In fact, projected variances on axis F1/F2 of the co-inertia analysis were close to the values of maximum projected variances on the 1^st^ and 2^nd^ axis of the PCA (Table 4). Comparison between the co-inertia coefficient RV and its empirical distribution during the Monte-Carlo test showed a strong co-structure between the two tables (RV = 0.239). Next, the test procedure was run on the two seasons separately, to check for a seasonal impact on the co-structure between benthos and pelagos. Results were more significant in spring (RV = 0.500), even if the summer RV (RV = 0.110) remained good (p<0.01 for the 3 tests).

**Table 4:** Comparison of inertia resulting from the separate analyses of each dataset.

Axis	InerBen	InerPel	InermaxBen	InermaxPel
F1	1.78	1.63	1.84	1.72
F2	1.02	0.738	0.967	0.731

Two co-inertia axes (F1 and F2) were selected. InerBen  =  inertia of the benthic table projected on co-inertia axes; InerPel  =  inertia of the pelagic table projected on co-inertia axes; InermaxBen  =  maximal projected inertia of the benthic table (1^st^ and 2^nd^ eigenvalue of the PCA); InermaxPel  =  maximum projected inertia of the pelagic table (1^st^ and 2^nd^ eigenvalue of the PCA).

**Figure 7:**
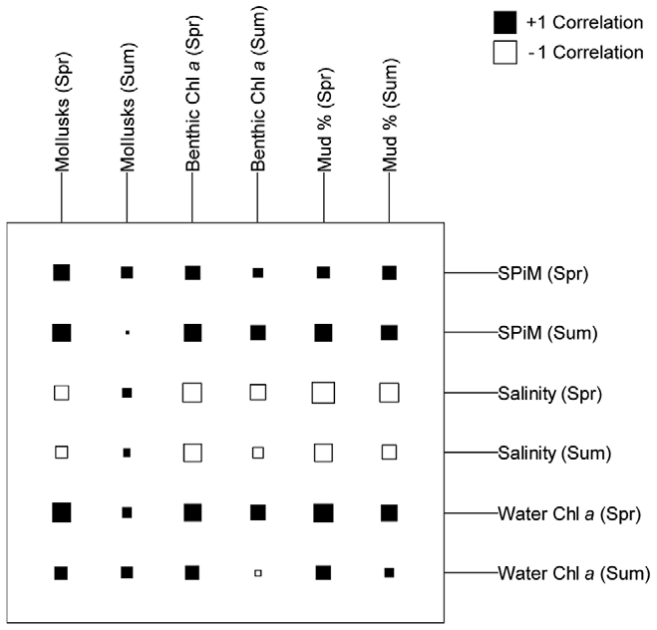
Cross-table resulting from the co-inertia analysis. Represents the correlation between the benthic and pelagic datasets.

The cross-table resulting from the co-inertia (Fig. 7) confirmed the strong impact of the season on benthos-pelagos coupling. In spring, mollusks exhibited a negative correlation with salinity, and a positive correlation with SPiM and water Chl *a* concentration. The levels of correlations were much lower than in spring data. Spring benthic Chl *a* concentration showed a negative correlation with salinity, whereas it showed a positive correlation with SPiM in both seasons. The mud fraction was positively correlated with water Chl *a* concentration in spring, and negatively correlated with salinity at the same period. The same relationship was found in summer, but to a lesser degree. Finally, in both season, the mud fraction was negatively correlated with bathymetry and positively correlated with SPiM.

Diatom taxonomic analysis revealed a number of taxa originating from different environments - marine, brackish and benthic - and characterized by different shapes and sizes (Table 5). Even if the species composition was almost the same at the two sampling periods, the relative proportions of the different species differed. The long-chain diatom *Asterionellopsis glacialis*, a brackish species observed at both sampling dates, was the dominant species in the Bay during the spring sampling (92.4%). The marine genus *Chaetoceros sp.* was dominant during the autumn sampling (73.4%) but was not identified in the spring samples. Benthic-pelagic ratios were calculated at several points distributed throughout the intertidal area, and represented on the Chl *a* concentration maps for both seasons (Fig. 5). At the first sampling date, the benthic-pelagic ratios were low throughout the Bay. However, benthic species reached 40% of the diatom community at two sampling points in the late summer sampling. Results showed that the benthic-pelagic ratios supported pelagic species over the entire map (Fig. 6), with percentages ranging between 60.2% and 100% in spring and between 59.6% and 100% except at two sites in summer. In fact, at these two sampling points, benthic diatoms species reached 47.0% and 47.2% of the diatom community. These two points corresponded to the area where the highest water Chl *a* concentration and SPiM were observed.

**Table 5:** List of determined microalgal taxa from Lugol fixed water samples.

Diatom species	Lifestyle	Shape	Size classes	Spring	Automn
*Asterionellopsis glacialis*	Tychopelagic	Pennate	Small (<15.10^3^ µm^3^.cell^–1^)	▪	▪
*Chaetoceros spp.*	Pelagic	Centric		□	▪
*Cyclotella spp.*	Pelagic	Centric		▪	▪
*Navicula spp.*	Benthic	Pennate		▪	▪
*Diploneis spp.*	Benthic	Pennate	Medium (15.10^3^ µm^3^.cell^–1^< *x*<150.10^3^ µm^3^.cell^–1^)	▪	□
*Gyrosigma fasciola*	Benthic	Pennate		▪	▪
*Gyrosigma hippocampus*	Benthic	Pennate		▪	▪
*Nitzschia longissima*	Benthic	Pennate		▪	▪
*Pleurosigma spp.*	Benthic	Pennate		▪	▪
*Pseudo-nitzschia spp.*	Benthic	Pennate		▪	▪
*Paralia marina*	Benthic	Centric		▪	▪
*Thalassionema nitzschioides*	Pelagic	Pennate		□	▪
*Thalassiosira rotula*	Pelagic	Centric		▪	▪
*Coscinodiscus wailesii*	Pelagic	Centric	Large (>150.10^3^ µm^3^.cell^–1^)	▪	▪
*Guinardia delicatula*	Pelagic	Centric		▪	▪
*Guinardia striata*	Pelagic	Centric		▪	▪
*Lauderia annulata*	Pelagic	Centric		▪	▪
*Odontella regia*	Pelagic	Centric		▪	▪
*Rhizosolenia imbricata*	Pelagic	Centric		▪	▪

Each species is classified by living, shape, size class and presence/absence during the two samplings.

**Figure 8:**
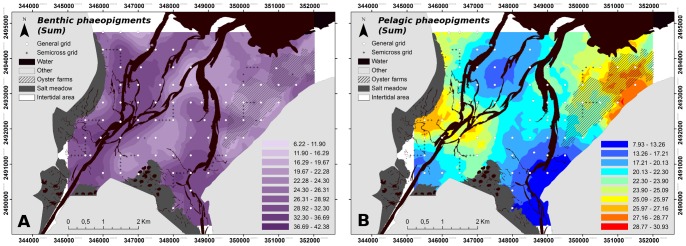
BDV summer kriged maps of both benthic (A) and pelagic (B) phaeopigments. Results are presented as % of total pigments.

The benthic phaeopigment percentage map (Fig. 8) showed a higher concentration close to the channels, and a lower concentration under the farming structures and in the central area between the two channels. The water column phaeopigment map showed a negative relationship with the water column Chl *a* concentration map (Fig. 6, 8), whereas no relationship was observed with the benthic phaeopigments map. The two depletion areas previously seen for the pelagic Chl *a* concentration were the two areas with the maximum phaeopigment percentages, ranging from 21.50% to 30.39% of the total pigments.

**Figure 9:**
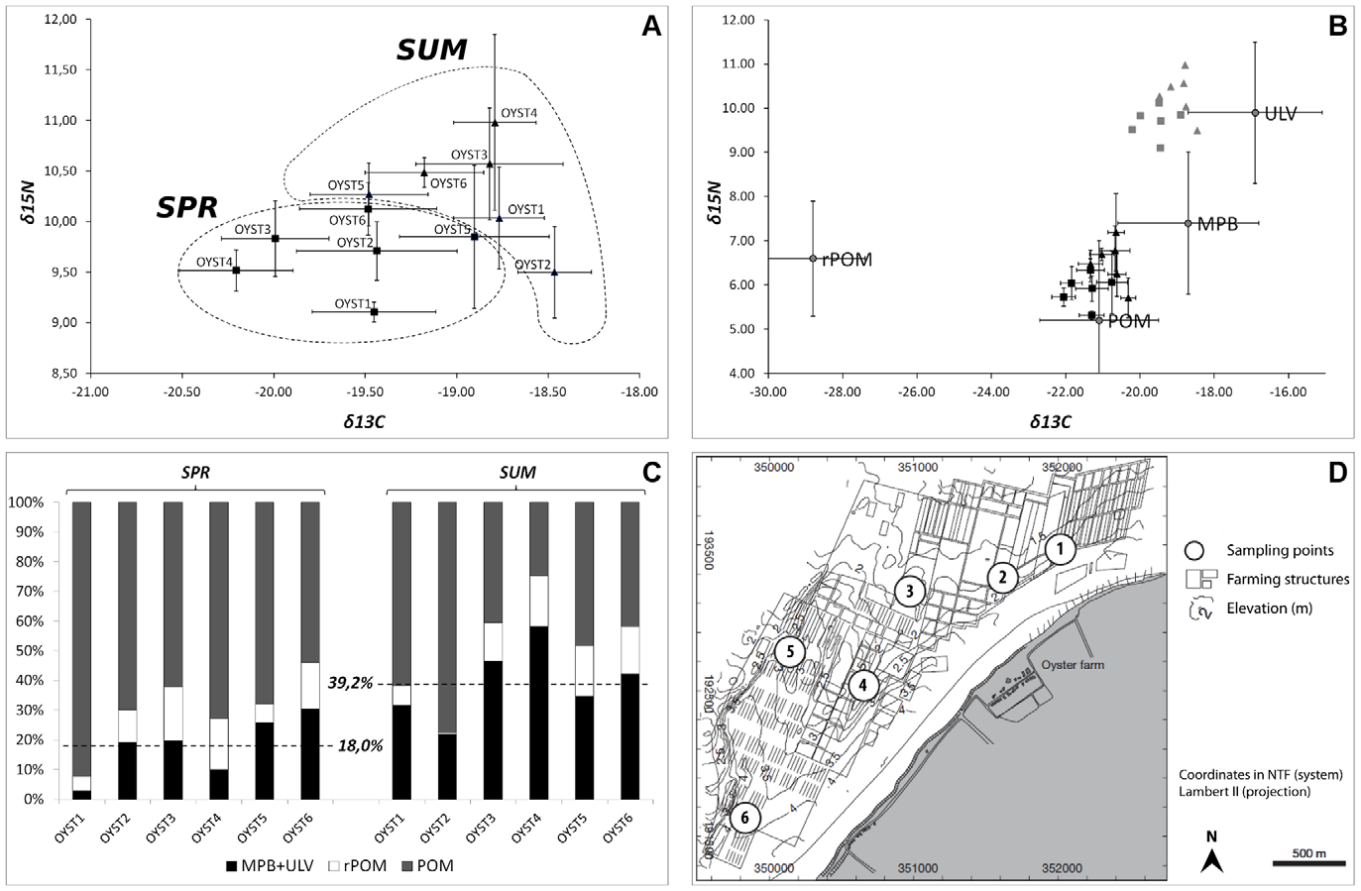
Temporal variations of *δ*13C and *δ*15N for *C. gigas* at 6 locations in the BDV. Isotopic signature (A), Isotopic signature before (gray) and after (black) fractionation (B), contribution of sources to oyster diets (C), location of oysters within the farming structures in the north-western part of the bay (D). Organic matter sources are plotted with standard deviations (see Lefebvre et al., 2009) to distinguish their relative contribution to the diets in the 2 sampling campaigns (B). Horizontal bars indicate the ±SD of the mean for n = 5.

Oysters sampled a month after each field campaign showed significant differences in isotopic signature (Fig. 9A). The *δ*_13_C values ranged between −20 and −19‰, and *δ*_15_N values between 9 and 10‰ in spring. These values increased in summer, ranging from −19 to −18‰ for *δ*_13_C and from 9.5 to 11‰. After correction of the trophic step fractionation (Fig. 9B), these values were clearly distributed between particulate organic matter (POM) and MPB. There was an increased contribution of MPB to the oyster diets (paired t-test, P-value  = 0.027), which increased from 18.0% in spring to 39.2% in summer on average (Fig. 9C). The spatial pattern of this contribution differed in the two seasons, with a decreasing south-to-north gradient in spring, whereas the maximum contribution was found at the OYST3 and OYST4 located the middle of the farming structures in summer (Fig. 9D). This area was found to be enriched in both mud and benthic Chl *a* concentration at the same season.

## Discussion

### MPB Spatial Distribution: Strong Effect of Mud Fraction and Bed Elevation

As shown in Orvain et al. [17] for this ecosystem, there was a clear relationship between MPB biomass and the grain-size of the sediment. Chl *a* concentration appeared to increase as a function of the mud fraction, in agreement to other studies ([52], [53]) which found higher Chl *a* content when expressed per mass unit. Moreover, both Chl *a* biomass and mud fraction were closely correlated with the bathymetry of the intertidal area, especially in area located on the west side of the Carentan channel. In fact, shallower water in areas with less hydrodynamic stress favored the silting up of these areas, and increased sunlight intensity, all of which favored MPB production [54]. Thus, in the present study, MPB biomass was well correlated to both mud fraction and bathymetry, as shown for other temperate estuarine ecosystems ([19], [55], [56]). In spite of the strong contrast between the two sampling periods in terms of temperature or light and nutrients availability, results revealed a perennial spatial structure of the intertidal sediments and MPB biomass in the bay (Fig. 4A, B, C, D) regarding to the stability of patterns between seasons at the year scale. The southern area close to the salt meadow was characterized by shallower waters, resulting in a muddy area because of the combination of direct river inputs and lower hydrodynamic conditions. Conversely, the northern part was under marine influence, with higher hydrodynamic conditions leading to sandy sediments. Finally, the eastern area appeared to be mainly influenced by the farming structures. The limited seasonal effect on the ranges of benthic Chl *a* concentration found in the BDV underlines the predominant effect of grain size and bathymetry on MPB distribution and biomass. As this bay is mostly made up of sandy sediments, these results correspond to those observed by van der Wal et al. [19], showing lower variability in sandy sediments than in muddy sediments. MPB distribution patterns were in close agreement with the results observed in April 2003 [17] even if the biomass levels were higher, certainly due to the much higher solar radiation observed during that exceptionally hot year.

### Mollusk Spatial Distribution: Direct Linkage with Water Chl a Spatial Patterns

Distribution patterns of mollusk biomass, mainly represented by *Cerastoderma edule*, appeared to be related to the water Chl *a* concentration for the two seasons. Distribution of macrozoobenthos in response to microphytobenthos and sediment has been studied by van der Wal [57] at an intertidal area scale, finding good model predictors to describe surface and deep deposit-feeders biomass using benthic variables such as MPB biomass or median grain-size, but the model found for suspension feeders was less satisfying with no predictor terms of the model significant. Honkoop et al. [58] also found low relationship between abiotic factors and the distribution patterns of benthos, suggesting that they could be influenced or determined by biotic interactions which may be more important than the assumed abiotic structuring they measured.

Our results confirm their suggestions about including pelagic variables to improve comprehension and modeling of macrofauna abundance or biomass distribution. If benthic variables may explain biomass and/or distribution of deposit-feeders, both benthic and pelagic variables must be assessed to explain better suspension-feeders biomass and/or distribution, underlining the necessity of including food compartment associated with studied communities. As a consequence the reverse reasoning has to be considered too when studying Chl *a* concentration in the water column, with higher cockle densities leading to higher bioturbation and consequently higher MPB resuspension.

### Benthic-pelagic Coupling: Impact of Resuspension Phenomenon

The same three part structure as for the benthic sampling was observed in the water column at two sampling seasons (Fig. 4G), showing on the one hand the fundamental influence of physical factors on benthic-pelagic coupling, and, on the other hand, its robustness over time in terms of both structure and resuspension phenomena. The pelagic structure is not perennial since water bodies are highly variable over time in terms of phytoplankton abundance and composition. As a consequence, the strong spatial structure of the benthic compartment influences the pelagic compartment through a domino effect controlled mainly by hydrodynamics and currents. Wind effect is well recognized as one of the first factor implicated in the temporal variation of resuspension phenomenon [4]. The results by de Jonge and van Beusekom focused on temporal dynamics in terms of resuspension phenomenon. By contrast, the present study was based on two samplings with similar hydrodynamic conditions but with a comprehensive number of stations to examine spatial patterns. Temporal detailed dynamics of the resuspension of microphytobenthos as well as wind effects were out of the scope of the present study which aims at describing spatial patterns of the benthic and pelagic variables without considering the wind. The results presented here and particularly the difference observed between the two samplings focus on other phenomena implicated in resuspension phenomenon. We must mention that contrary to more open estuarine ecosystems, BDV is relatively protected of wind effects by the geographical configuration of this basin, which is protected by southern and/or western dominant winds by the Cotentin Peninsula. Only northern (and especially north-eastern) winds can have an impact on the general functioning of this bay in terms of erosion.

The pelagic Chl *a* concentration was closely correlated with the concentration of the SPiM, but the low or null relationship with salinity revealed the influence of resuspension events rather than river inputs. In fact, the two channels were characterized by low SPiM concentrations (Fig. 6E and F). Moreover, both Chl *a* concentration and SPiM concentration were inversely correlated with bed elevation (Fig. 5A), reinforcing the hypothesis of resuspension events from the muddier sediments with a higher impact in these shallow waters [59]. Similarly, good levels of correlation between SPiM and Chl a in the water column were obtained by Guarini et al. during large-scale [60] and long-term [48] samplings in another estuarine bay (Marennes-Oléron) where microphytobenthic communities are more developed than in BDV.

The significant co-structure found between the benthic and pelagic compartments confirms the hypothesis of a strong coupling, maintaining the 3-part structure in both compartments and at both seasons. The dominance of *Asterionelopsis glacialis* and *Chaetoceros spp.* during the respective sampling periods in this ecosystem has already been reported in the literature [61]. It reflects changes in the estuarine microalgae communities over the year, with dominance of brackish species in early spring and of marine species in late summer. However, resuspension phenomena appear to be relatively stable, given the range of the MPB ratios revealed by taxonomic identification. Only two locations showed a high MPB ratio during the late summer sampling, corresponding to a higher SPiM and an area of lower current velocities observed at the same period. According to the benthic:pelagic ratio of this patch and to microscopic observations, a part of this resuspended MPB is probably the result of inputs from the eastern channel. However, taxonomic analyses have to be interpreted with caution. The distinction between plankton and benthos is not perfectly clear because some microalgae are tychopelagic, i.e. they live in both environments [62]. Actually, in the surf zone (the zone extending from the outermost line of breakers to the limit of wave uprush) communities dominated by long chain diatoms like *Asterionellopsis glacialis* can be deposited on the sediment by the ebb tide, because mucus and particles attached to the cells increase their density, hence increasing sedimentation [63]. As a consequence, the number of living *A. glacialis* cells per sediment area behind the surf zone can be on average four orders of magnitude higher than the concentrations found in the respective water column [64].

The mollusk biomass was 20-fold higher in summer than in spring and macrofaunal activity is also known to increase between spring and summer because of the effects of temperature, so higher biological activity at the bay scale resulted in a higher phytoplankton consumption and subsequently in an increase in biodeposition. This high consumption rate was confirmed by phaeopigments released in the water column (Fig. 8B). Finally, the isotopic signature of the diatom *Asterionellopsis glacialis* needs to be investigated since it could regulate its buoyancy in order to stay in the bay [65], since it belongs both to benthic and pelagic environment. Its presence, highest in spring, could help explain the good overlay of the benthic and pelagic maps.

In spring, resuspension phenomena were mainly under hydrodynamic influences resulting in an almost complete overlaying of benthic and pelagic compartment map, reflecting the close coupling between them. In summer, the increase in mollusk biomass increased the effects of bioturbation that could be involved in different chl *a* fluxes like consumption, biodeposition and bioresuspension processes. The role of bioturbators is well known as an important factor controlling microphytobenthos resuspension [13] [66]. Macrofauna activities and especially those of the cockle *Cerastoderma edule* must be better explored and evaluated because our results suggest that this is, when not considering wind effect, the prime factor controlling resuspension rates of microphytobenthos at the scale of the bay. This process must be thoroughly involved in the good relationship between suspension-feeder biomass and concentrations of resuspended chl *a*.

### Impact of Cultivated Oysters in the Benthic-pelagic Coupling

The intertidal area is divided into two parts with respect to the Isigny channel, the two parts being clearly separated by the presence/absence of oysters farming structures. The spatial distribution of studied variables in the eastern part of the bay allows deciphering of the oyster impact on the benthic-pelagic coupling. Even if this area was characterized by sandy sediments with a very small mud fraction and a high depth, MPB biomass was high. Several explanations are possible and/or a combination of them.

First, microalgae communities can benefit from biological phenomena such as biodeposition under the farming structures, providing a favorable habitat for MPB assemblages [67]. Increased oyster filtration activity in late summer [68] led to a higher biodeposition, explaining two little patches with a higher mud fraction in the east in late summer (Fig. 4D) and the high phaeopigment percentages in the water column (Fig. 8B). Nevertheless, biodeposition was a local phenomenon which was mostly visible under the farming structures, and may be insufficient to significantly increase the mud fraction of the sediment at the scale of the eastern area. The two small patches could be explained by a combination of cultivated stocks, currents or bathymetry difference over farming structures [69]. Secondly, the high clearance rate of oysters significantly affected light availability by reducing water turbidity [18], thus enhancing MPB production.

Thirdly, the eastern area was characterized by very low concentrations of wild mollusks and biomass, in contrast to the western part of the bay. The exclusion of wild suspension-feeders under farming structures has already been observed at this site by Dubois et al. [70], showing that there was a shift in the trophic chain to high levels, with a predominance of predators, especially under farming structure. The primary consumption rate of MPB by grazers must therefore be low under the farming structures.

Finally, this part of the bay could be dominated by epipsammic species, explaining the contrast between the null or low mud fraction and the high Chl *a* concentration in both spring and summer. Sandy sediments have been reported to show more diverse assemblages than muddy sediments, including epipsammic diatoms, euglenids and cyanobacteria [71].

Porter et al. [25] found that a low degree of tidal resuspension is also responsible for a general shift from phytoplankton primary production to microphytobenthic primary production (by a cascade of effects where light and nutrient availabilities are also involved). Such effects must be implicated in the ecological functioning of this farming zone and this general shift must be still reinforced by the biodeposition fluxes due to oysters. Among the 4 hypotheses mentioned above for explaining the high concentrations of MPB biomass in this zone, none of them can be really excluded. A combination of all these processes must interfere in interactions with tidal hydrodynamics, and it appears very delicate to disentangle the relative contribution of each of these processes. The reduction in pelagic Chl *a* concentration observed above the farming structures highlighted the filtration efficiency of oysters, which was also confirmed by the percentage of water phaeopigment. The latter variable provides an argument in favour of the direct production of pseudofeces after consumption of microalgae and/or resuspension events of easily erodible sediments with high biodeposits under the farming structures [66]. However, the lack of match between benthic phaeopigments and both pelagic Chl *a* concentration and water phaeopigments suggests that it resulted mostly from the direct consumption of microalgae. These observations confirm the adequacy of the model proposed by Grangeré et al. [69], which represent ecosystem functioning with or without the presence of oysters, and revealed the prevailing effect of their top-down regulation in this area.

Since mollusk biomass was 20 times higher in summer than in spring, primary consumption would be expected to be higher in summer. However, the relative constancy of the MPB biomass levels between the two seasons suggests that the primary consumption is balanced by a higher primary production in summer. The eastern part of the bay was characterized by the absence of wild mollusks under farming structures, confirming the exclusion of suspension feeders already observed by Dubois et al. [70]. Suspension feeders may be disturbed by both biodeposition [72] and/or overconsumption of organic matter by the oysters. Thus, the lack of a correlation between the spatial patterns of MPB and macrofauna can be mainly attributed to the fact that most of the mollusk biomass was made up of suspension feeders and especially of *C. edule*, which widely dominated the mollusk assemblage, rather than deposit feeders that feed exclusively on MPB.

### Impact of Resuspension for Higher Trophic Levels: Evidence for Allochtonous Feeding

Isotopic signatures showed that oysters consumed 2 times more MPB in summer than in spring, leading us to formulate three hypotheses: i) local feeding of autochtonous MPB directly associated with resuspended biodeposits under farming structures, ii) reduced phytoplankton abundance in late summer compared to spring, leading to a higher relative abundance of MPB in the potential food pool, iii) a higher resuspension at the bay scale and especially from the adjacent area in the south, that consequently supplies trophic resources to the cultivated oysters [73]. Regarding the limited differences between the two seasons in terms of benthic Chl *a* concentration, the first one can be ignored. Moreover, fluxes would not be expected to be very different since similar hydrodynamics and mollusk densities were found at both seasons under the farming structures, supporting the hypothesis of an allochtonous feeding of oysters. At 1 m depth Chl *a* concentration levels are similar in April and September, so that the second hypothesis seems unlikely. Taxonomic analyses revealed higher benthic-pelagic ratios for the 2 samplings in the adjacent area of the forming zone at the south/west, reinforcing the third hypothesis, which seems to be the most reliable one, when merging all data. A higher resuspension rate at the bay scale results from a change in the forcing variables, meaning one of the following compartments: hydrodynamics, sediment properties and/or macrofauna. Both samplings campaigns were conducted during spring tides with similar hydrodynamic conditions, and sediment properties revealed only slight differences between the two seasons. Higher resuspension could be explained by the huge increase in mollusk biomass between the two seasons. In fact, the cockles C*erastoderma edule* have been shown to increase resuspension phenomena via bioturbation [74]. Therefore, the better coupling between benthic and pelagic compartments in spring can probably be explained by a predominance of physical factors. The increase in mollusk biomass/activity in summer drastically altered the balance between benthic and pelagic Chl *a,* with an increased role for biological factors in resuspension phenomena.

### Conclusion

To better assess resuspension phenomenon at an ecosystem scale, a special effort was made to study benthic and pelagic variables at the same time, to better unravel causes of resuspension between biotic and abiotic factors. This *in situ* study is the first to analyze benthic-pelagic coupling at a bay scale in terms of body masses advection and trophic routes. The spatial heterogeneity of this ecosystem enabled the predominant physical and/or biological processes to be highlighted as a function of the area and/or season. The perennial structure observed at the scale of the whole bay provides evidence for the significant involvement of resuspension phenomena at the bay scale. Although physical factors appeared to predominate during winter/spring, in summer, biological factors can significantly increase exchanges between benthic and pelagic compartments when not considering the wind. The use of a multicriteria approach (robust approach plus unusual indicators) to trophic/taxonomic indicators made it possible to strongly suggest a role for resuspension and benthic zonation in the spatial distribution of Chl *a* concentration in the water columns in two contrasting seasons and also that mollusks and particularly the cockle *Cerastoderma edule* play a role in microphytobenthic resuspension and its availability for oysters (*Crassostrea gigas*). When the biomass of these mollusks increases too much, this positive effect is masked by a high consumption rate leading to local depletion of Chl *a* concentration and SPiM. In fact, mollusk spatial distribution has a direct linkage with water Chl *a* concentration spatial patterns, which might have a structuring role on suspension-feeders. This highlights the fact that it is of critical importance to consider the connection between adjacent areas in terms of trophic relationships and microphytobenthos advection for farming structure management. These results underline the importance of taking biological phenomena into account in benthic-pelagic coupling to better evaluate the impact of resuspension for higher trophic levels.

Our study clearly suggests that there is not only a direct resuspension of microphytobenthos from the south of the bay but also an exportation from the water body of this habitat to another one at the north, then supplying food items to the cultivated oysters of the bay. Such trophic connections between adjacent habitat is of prime importance to consider because ecosystem models must consider these processes from the primary benthic production to the final consumption by suspension-feeders by including resuspension and advection in order to evaluate the real contribution of these areas as potential sink/sources of carbon [60].
